# Assessment of local pulse wave velocity distribution in mice using k-t BLAST PC-CMR with semi-automatic area segmentation

**DOI:** 10.1186/s12968-017-0382-2

**Published:** 2017-10-16

**Authors:** Volker Herold, Stefan Herz, Patrick Winter, Fabian Tobias Gutjahr, Kristina Andelovic, Wolfgang Rudolf Bauer, Peter Michael Jakob

**Affiliations:** 1 Department of Experimental Physics, University of Würzburg, Am Hubland, Würzburg, 97074 Germany; 2 Department of Internal Medicine I, University Hospital Würzburg, Würzburg, Germany; 3 Comprehensive Heart Failure Center/Deutsches Zentrum für Herzinsuffizienz, University of Würzburg, Würzburg, Germany

**Keywords:** Pulse wave velocity, Magnetic resonance imaging, Phase contrast, ApoE ^(−/−)^

## Abstract

**Background:**

Local aortic pulse wave velocity (PWV) is a measure for vascular stiffness and has a predictive value for cardiovascular events. Ultra high field CMR scanners allow the quantification of local PWV in mice, however these systems are yet unable to monitor the distribution of local elasticities.

**Methods:**

In the present study we provide a new accelerated method to quantify local aortic PWV in mice with phase-contrast cardiovascular magnetic resonance imaging (PC-CMR) at 17.6 T. Based on a k-t BLAST (Broad-use Linear Acquisition Speed-up Technique) undersampling scheme, total measurement time could be reduced by a factor of 6. The fast data acquisition enables to quantify the local PWV at several locations along the aortic blood vessel based on the evaluation of local temporal changes in blood flow and vessel cross sectional area. To speed up post processing and to eliminate operator bias, we introduce a new semi-automatic segmentation algorithm to quantify cross-sectional areas of the aortic vessel. The new methods were applied in 10 eight-month-old mice (4 C57BL/6J-mice and 6 ApoE ^(−/−)^-mice) at 12 adjacent locations along the abdominal aorta.

**Results:**

Accelerated data acquisition and semi-automatic post-processing delivered reliable measures for the local PWV, similiar to those obtained with full data sampling and manual segmentation. No statistically significant differences of the mean values could be detected for the different measurement approaches. Mean PWV values were elevated for the ApoE ^(−/−)^-group compared to the C57BL/6J-group (3.5 ± 0.7 m/s vs. 2.2 ± 0.4 m/s, *p* < 0.01). A more heterogeneous PWV-distribution in the ApoE ^(−/−)^-animals could be observed compared to the C57BL/6J-mice, representing the local character of lesion development in atherosclerosis.

**Conclusion:**

In the present work, we showed that k-t BLAST PC-MRI enables the measurement of the local PWV distribution in the mouse aorta. The semi-automatic segmentation method based on PC-CMR data allowed rapid determination of local PWV. The findings of this study demonstrate the ability of the proposed methods to non-invasively quantify the spatial variations in local PWV along the aorta of ApoE ^(−/−)^-mice as a relevant model of atherosclerosis.

## Background

Arterial stiffness (AS) assessed by pulse wave velocity (PWV) has been shown to represent a valuable biomarker of cardiovascular disease (CVD) risk [1, 2]. PWV is considered to be the most validated method for noninvasive quantification of AS [3]. It has been shown that carotid-femoral PWV as the gold standard method for determination the arterial stiffness is associated with higher CVD events in high risk [4] and community-based samples [5]. PWV is of special interest in the aorta, since AS outside the aortic track has limited predictive value, as shown in patients with end-stage renal disease [6].

Propagative models consisting of a flexible tube are widely used to describe basic mechanical properties of central arterial vessels such as a finite traveling wave speed known as the PWV. Regional PWV is considered the gold standard measure of AS, given its strong prediction of adverse outcomes [3]. It is generally measured using the so called transit-time (TT) method. This technique requires the recordings of two pulses (such as pressure-, flow- or distension-pulses) sampled with a common time base at different locations along the blood flow traveling pathway. Identifying the temporal shift, *Δ*t, among the pulses allows the calculation of the regional PWV, given as the traveling distance *Δ*s divided by the traveling time *Δ*t.

Depending on the type of recorded cardiac pulses, different modalities can be utilized to determine the PWV such as mechanotransducers sampling the pressure pulse [7] or Doppler probes measuring blood flow [8]. Although widely used, both methods are limited by inaccurate estimation of the distance *Δ*s, since the pulse traveling pathway is not directly accessible.

Cardiovascular magnetic resonance imaging (CMR) has several advantages over these methods: Pulse traveling distances can be measured precisely, since a three dimensional visualization of the vessel is possible, which also increases the reproducibility of locations in long term studies. Furthermore, imaging planes can be oriented perpendicular to the flow direction, increasing accuracy in determining vessel distensibility. Since phase-contrast cardiovascular magnetic resonance imaging (PC-CMR) allows the simultaneous acquisition of vessel wall morphology and blood flow, CMR is well suited to measure local parameters related to arterial stiffness such as distensibility and local PWV [9, 10].

Apolipoprotein E-deficient (ApoE ^(−/−)^)- mice are considered to be an important model of atherosclerosis, since they develop atherosclerotic lesions of morphology similar to those observed in humans [11, 12]. CMR and echocardiography have proven to be valuable techniques to investigate the evolution of arterial stiffening caused by atherogenesis in mice. Most of these techniques are limited to the evaluation of the regional PWV based on TT measurements and thus revealing only the mean stiffness of the included vessel segment [13–16].

Elastic properties of conduit arteries are known to vary from proximal to distal [17]. In early atherosclerosis, lesions and subtle alterations in molecular and cellular structure are distributed heterogeneously along the vessel wall. Typical locations for early plaque development emphasize the need for the assessment of local elasticity parameters [18]. Invasive examinations in human coronary arteries have shown that local and global coronary PWV can be different, in particular under vasodilation and hyperemia [19].

Non-invasive CMR-techniques for the determination of the local PWV, like the Flow/Area (QA)-method became recently available and have shown to be more sensitive in revealing early atherogenetic changes than shown by morphological thickening [20–22].

These new techniques can only deliver local PWV values for one or two different locations within one examination and are therefore unable to monitor the distribution of local elasticities.

However, the full-sampled CINE-datasets comprise a high degree of data redundancy in the temporal dimension, making it particularly suitable to acceleration techniques such as k-t BLAST (Broad-use Linear Acquisition Speed-up Technique) [23]. The PWV is calculated on the basis of mean linear temporal changes of the blood volume flow as a function of the cross sectional area. Therefore high temporal frequency components are less important, allowing for a high acceleration factor. The PWV calculation is less vulnerable to a high overlap of the point-spread functions in the xf-domain leading to temporal blurring [24, 25].

In the present work we propose an advanced method based on the QA-technique and 6-fold accelerated PC-CINE-CMR with k-t BLAST, facilitating the acquisition of a series of adjacent slices to determine the local PWV distribution along a certain vessel wall segment. To dispense with time consuming manual segmentations, we secondary propose a new semi-automatic segmentation technique to determine cross sectional area changes.

The new methods were compared to a previously presented method and subsequently applied to two groups of ApoE ^(−/−)^- and C57BL/6J-mice, respectively [21].

## Methods

### PC-CMR pulse sequence

**Fig. 1:**
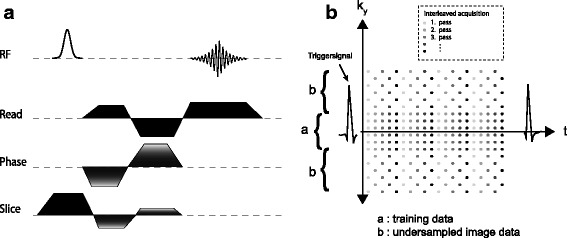
**a** 2D-Phase-Contrast-CINE-FLASH sequence with through-plane flow encoding. Flow-compensation was applied for all three-gradient directions. **b** Undersampling scheme for the k-t BLAST data acquisition (shown for *R*=2). The different shadings of the data points represent the different repetitions of the sequence. Altogether five repetitions, each one shifted by a temporal delay of 1 ms were, performed

All measurements were performed using a 17.6 T ultra high-field system (Bruker Avance 750 WB, Bruker BioSpin MRI GmbH, Rheinstetten, Germany), equipped with a 1 T/m gradient system and a home-built radio frequency (RF)-resonator in birdcage design (inner diameter: 27 mm). A series of 2D-FLASH localizers was applied in advance to navigate to the abdominal aorta. To measure the time course of the blood volume flow Q and the cross sectional area A, a high resolution PC-CINE-FLASH sequence was performed perpendicular to the abdominal aorta with through plane flow encoding as shown in Fig. 1 a. Two flow encoding datasets (first gradient moment *M*_1_ = ± 0.3 s/m) and one flow compensated dataset were acquired one at a time, to extract voxel-wise blood flow velocity values. Velocity information for each pixel was calculated by fitting a line to the phase data as a function of the first moments of the velocity encoding gradients. Acquisition of three instead of two different flow encoding steps allowed to estimate the error of the linear fit used to determine the velocity data. Velocity values based on data points with *R* ^2^<0.85 (R: correlation coefficient) were excluded from further calculations, as described in [21]. A temporal resolution of 1ms was achieved by a time shifted repeated acquisition of 5 CINE datasets, each at a native temporal resolution of TR =5 ms, as described in [21]. Further imaging parameters were: TE =2.1 ms, FOV =22 × 22 m*m* ^2^, matrix =150 × 150, slice-thickness =0.7 mm, final resolution after zerofilling 86×86μ*m* ^2^, total acquisition time per slice: approx. 1 min.

### k-t BLAST Acceleration

**Fig. 2:**
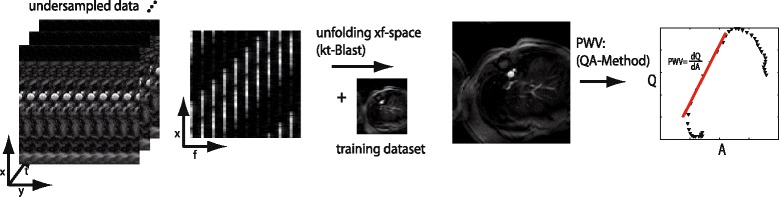
Post processing steps for the k-t BLAST QA-reconstruction

K-t BLAST acceleration was implemented according to [23]. Fig. 1 b schematically shows a lattice sampling pattern for an acceleration factor *r*=2. For in vivo measurements, data acquisition was accelerated by *r*=10, supplemented with a training data set of 10 centered k-space lines, leading to an effective acceleration factor of 6. The post processing of the undersampled data is described in Fig. 2. After transferring the folded 3D dataset (x,y,t) into the xf-space, the data were unfolded by using a low-resolution training dataset according to the k-t BLAST reconstruction scheme. The process of unfolding data in xf-space can be described mathematically as filtering the aliased data [23]:

1 $$ \boldsymbol{\rho}=\boldsymbol{M}^{2}\boldsymbol{1}^{H} \left(\boldsymbol{1}\boldsymbol{M}^{2}\boldsymbol{1}^{H}\right)^{-1} \rho_{alias}  $$

where *M* is a diagonal matrix with shifted versions of the undersampled training data sets along the diagonal. The resolution of the training dataset (i.e. the number of center-k-space lines) regulates the high-temporal frequency suppression in the reconstructed images (by spatially averaging high frequency effects), acting like a low pass filter on the final QA-plots. By adjusting the number of center-k-space-lines to 10 in the training dataset we could mimic the effect of the originally applied low pass filters on the QA-data [21]. Thus, the total measurement time could be reduced by a factor of 6 without compromising the accuracy of the PWV calculation.

### Local PWV calculation

If time dependent data for the blood volume flow Q and the aortic cross section A are available, the local PWV can be calculated as shown in [10]:

2 $$ PWV=\frac{dQ}{dA} \,.  $$

This equation holds for early systolic time-points, when the flow pulse can be considered as unidirectional and reflection-free. Thus, in order to calculate the PWV, the Q(A)-curve was fitted to a line for time-points belonging to the early upstroke of the systolic flow pulse. In general, Q(t) can be measured with higher precision than A(t), since the parabolic flow profiles minimize deviations near vessel boundaries, where segmentation artefacts might occur. However, accurate cross sectional area segmentation is crucial at the limited spatial resolution, since small deviations in segmentation results can significantly distort the PWV calculation. For manual segmentation it has proven to be useful to apply a low pass filter on A(t) before calculating the PWV [21].

### Segmentation

**Fig. 3:**
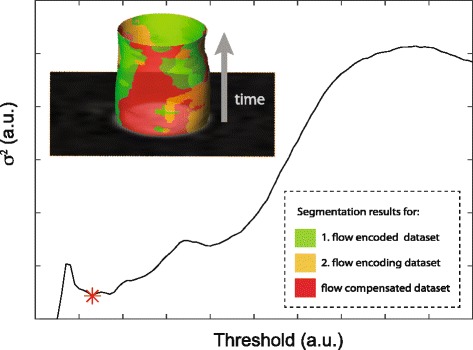
Semi-automatic segmentation of the cross sectional area of the vessel lumen. For a given threshold the time course of the cross sectional area was automatically determined for each of the three flow encoding steps. The threshold delivering the minimal deviation between the three segmentation results was considered as the optimum result

Manual segmentation of the cross sectional area, as done in [21, 22], can be very time consuming and data accuracy and reproducibility can be highly dependent on the operator. In the present work, we introduce a classification method incorporating prior knowledge about the measurement process. The basic principle is to involve magnitude data from all three motion encoding data sets into the segmentation process. The segmentation is based on the assumption that the magnitude images of each of the three motion encoded data sets should lead to the same segmentation result. The segmentation process can then be formulated as an optimization problem:

3 $$ \underset{{\tau}}{\arg\min} \left\{ \operatorname{E}\left[\left(\boldsymbol{\mathcal{P}}(\tau) - \operatorname{E}[ \boldsymbol{\mathcal{P}} (\tau)]\right)^{2}\right] \right\}  $$

where $\boldsymbol {\mathcal {P}}(t)=(\mathcal {P}_{1}(\tau), \mathcal {P}_{2}(\tau), \mathcal {P}_{3}(\tau)) $ gives the number of identified pixels in the binary masks for each flow encoding step when applying a certain threshold *τ*. The cost function represents the variance *σ* ^2^ of the threshold segmentation for the different flow encoding steps. The threshold value belonging to a minimum deviation between the flow encoding steps was found by evaluating the cost function for a given range of threshold values and numerically finding the minimum. Figure 3 shows the course of *σ* ^2^ as a function of threshold *τ* and the representative segmentation results for the different flow encoding steps. Cross sectional area for each time frame was subsequently calculated as $ \bar {\mathcal {P}} \times $ Voxel_Size.

### In vivo measurements

The proposed methods were applied in two mouse groups, both at the age of 8 months (ApoE ^(−/−)^ and C57BL/6J-mice (*n*=6, *n*=4 respectively)) and compared to the method published in [21]. 12 adjacent slices were acquired in the abdominal aorta. To evaluate the accuracy of the semi-automatic area segmentation, cross sectional-areas for three animals (two C57BL/6J-mice an one ApoE ^(−/−)^-mouse) at altogether 19 positions along the abdominal aorta were determined with manual and semi-automatic segmentation. Manual segmentation was done using the magnitude data of the flow compensated dataset.

Animals were anesthetized using isoflurane ((1.5–2 Vol. %) and 0_2_ (2L/min)). The mouse temperature was kept constant at 37 °C during the MR measurements by adjusting the temperature of the gradient cooling unit. All experimental procedures were in accordance with institutional and internationally recognized guidelines and were approved by the Regierung von Unterfranken (Government of Lower Franconia, Würzburg, Germany) to comply with German animal protection law. The reference number of the permit of the animal experiments is 55.2-2531.01-23/11.

### Statistical analysis

Bland-Altmann analysis and a Student’s paired t-test were performed to verify the agreement between fully sampled local PWV-measurements and the k-t BLAST acceleration. Statistical significance was defined as *p* <0.05. A sample size calculation for a paired two tailed t-test yielded a sample size of n=10, with mean difference *Δ* *μ*=0.5 m/s (expected difference), *σ*=0.5 m/s (standard deviation), *p*=0.05, 1−*β*=0.8 (power).

Semi-automatic area segmentation and manual area segmentation were compared by examining 760 (19*40) aortic vessel cross sections. Both, manual segmentation at a low resolution (86×86) *μ* *m* ^2^ (low level of zerofilling) and at a high spatial resolution (21×21) *μ* *m* ^2^ were compared with the semi-automatic segmentation approach (resolution: (21×21) *μ* *m* ^2^) to evaluate the influence of partial volume effects. A linear regression was performed to identify the concordance between the different segmentation approaches. Bland-Altmann analysis and a paired t-test were performed to identify differences in mean values of the obtained PWV measures.

T-test was also performed to compare PWV-mean values of the ApoE ^(−/−)^-animal group and the C57BL/6J-animal group.

## Results

### k-t BLAST-acceleration

**Fig. 4:**
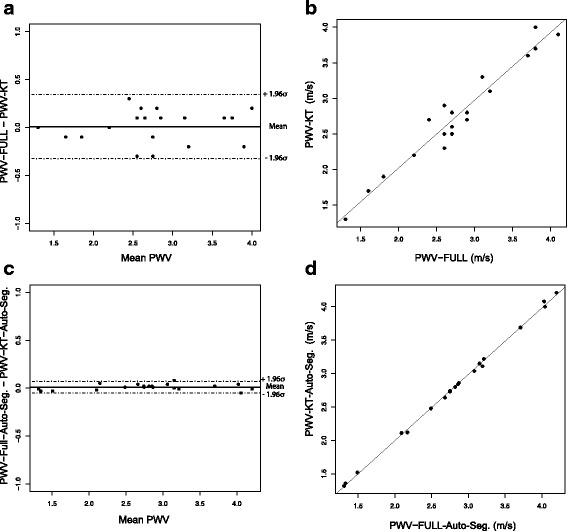
Bland-Altman plot (**a**) and scatter plot (**b**) of pulse-wave velocities obtained with k-t BLAST acceleration compared to PWV-measures from fully sampled datasets based on manual segmentation of the vessel cross section. **c**, **d** Bland-Altman plot and scatter plot of full sampled data versus undersampled data based on semi-automatic segmentation of the vessel cross section

To verify the agreement between fully sampled local PWV-measurements and the k-t BLAST acceleration, both measurements were applied at two randomly chosen locations along the abdominal aorta in each animal of the ApoE ^(−/−)^ and the wild-type group, respectively. All cross sectional areas were segmented manually and semi-automatically. In Fig. 4 a/b the results of both acquisition techniques are compared (using manual segmentation), illustrated by a Bland-Altman plot and a scatter plot. No systematic drift over the whole range of PWV-values is recognizable (Pearsons *r*=0.98, slope m ± 95% confidence interval: m =1.02±0.11). Both methods deliver PWV-values with corresponding arithmetic mean values.

Since the semi-automatic segmentation is not dependent on an operator input, when using this technique, there is almost no detectable difference between the accelerated and the fully sampled measurement as shown in Fig. 4 c/d (*r*=1, m =1.00±0.02).

Repeated measurements of the local PWV at one fixed location (*n*=7) with fully sampled data acquisition (not shown in Fig. 4) yielded a standard deviation of 0.2 m/s, which approximately corresponds to the deviation when comparing fully sampled local PWV-measurement and k-t BLAST acceleration.

### Semi-automatic area segmentation

**Fig. 5:**
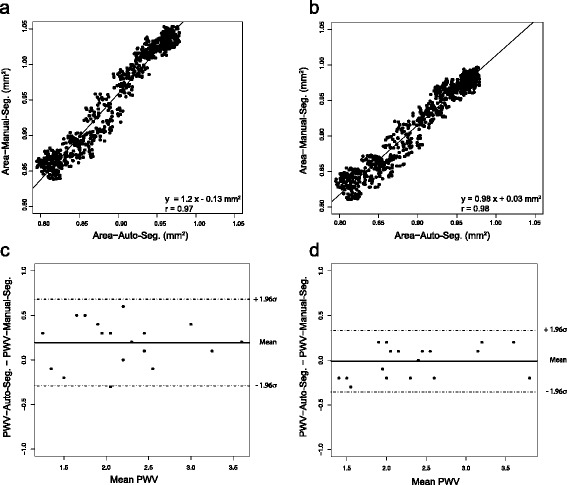
**a**, **b** Scatter plots of the cross sectional area segmentation: manual versus semi-automatic. **a** Spatial resolution achieved by zero padding for manual segmentation: (86×86) *μ* *m* ^2^; semi-automatic segmentation: (21×21) *μ* *m* ^2^. **b** Same spatial resolution for manual and semi-automatic segmentation. **c**, **d** PWV calcualtions based on the data shown in **a**, **b** respectively

Fig. 5 a shows a scattered plot of the manual segmentation versus the semi-automatic segmentation. The manual and semi-automatic segmentation methods demonstrated high agreement, but a significant bias towards higher cross-sectional areas using the semi-automatic method (Pearson *r*=0.97, y=1.2 x - 0.13 mm^2^).This bias can also be found in the PWV calculation. Figure 5 c shows the corresponding PWV-values. A small but significant difference between the mean values can be detected (paired t-test: *p* < 0.01).

For the data presented in Fig. 5 a/b, data were zero filled to different levels of resolution. Manual segmentation was performed at a resolution of (86×86) *μ* *m* ^2^, whereas for the semiautomatic segmentation data were zero filled ending up with a resolution of (21×21) *μ* *m* ^2^. Edge-detection with low resolution data might lead to an overestimation of the cross section, in particular for late systolic time-frames when the inflow-effect leads to a strong signal enhancement. To examine if the vessel cross section was overestimated for late systolic time-frames due to lower spatial resolution, the entire manual segmentation of the 19 positions was repeated at the same high spatial resolution as used or the semi-automatic segmentation.

Figure 5 b shows the corresponding scatter plot. For the high resolution comparison manual and semi-automatic segmentation methods showed a high agreement, without biased data towards higher cross-sectional areas (Pearson *r*=0.98, y=0.98 x + 0.03 mm^2^). Successively, the PWV values were calculated based on the temporal changes of flow and cross sectional areas, respectively. The results are shown in Fig. 5 d. There is no significant difference of the mean values of the calculated PWV values by manual and semi-automatic methods.

### In vivo study

**Fig. 6:**
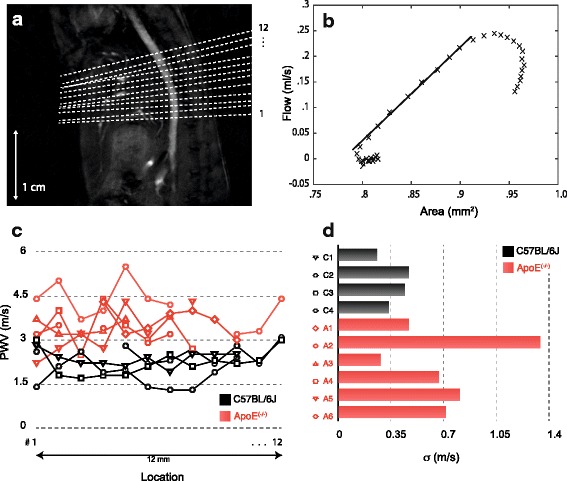
Results of the *in-vivo* measurements: **a** Distribution of the measurement locations to estimate the local PWV in the abdominal aorta. **b** Exemplary determination of the PWV for one measurement slice. **c** Distribution of the local-PWV measures for wild-type and ApoE ^(−/−)^-animals. **d** Standard deviation of the set of PWV-measures for each animal

Figure 6 shows the results of the in vivo measurements. PWV was measured at 12 contiguous locations along the abdominal aorta as shown in Fig. 6 a. For each location, blood flow and cross sectional area changes were measured and successively the PWV was calculated as depicted in Fig. 6 b. An overview of the distribution of the single PWV-measurements can be found in Fig. 6 c. Close to the diaphragm in the region of the upper abdominal aorta and in the vicinity of vessel branches, the vessel wall borders could not be detected reliably and thus prevent an accurate estimate for the local PWV. For these cases no data points are shown in Fig. 6 c. In particular with animals from the ApoE ^(−/−)^-group, magnitude images often were corrupted in the upper part of the abdominal aorta due to flow artefacts caused by a varying heart rate (altogether 26% of the slices were excluded from the PWV calculation). However, if evaluating mean values for each animal group, the results yield a significant (p < 0.01) elevated PWV for the ApoE ^(−/−)^-mice. Mean PWV for the ApoE ^(−/−)^-group was measured to be (3.5 ± 0.7) m/s (mean ± standard deviation), whereas wild-type mean PWV was (2.2 ± 0.4) m/s. When considering the individual distributions of the local PWV, no formative patterns for the local elasticity could be identified in neither animal group. PWV-values in the ApoE ^(−/−)^-group however, are subject to a significant stronger form of individual fluctuations along the flow path way. This can be seen when evaluating the standard deviations for the PWV values in each animal, as shown in Fig. 6 d.

## Discussion

In the present study, we introduce an improved and accelerated approach to assess the local PWV in the mouse abdominal aorta with PC-CINE-CMR. K-t BLAST acceleration allowed for data acquisition 6 times faster than with fully sampled data. K-t undersampling however, comes with the drawback of a reduced temporal resolution after reconstruction. This effect similiarly can be achieved by applying a temporal low pass filter to the final flow and cross sectional area data, respectively, as done in [21]. Instead of using k-t BLAST-CMR, one could also imagine to acquire fully sampled data without the proposed interleaved acquisition scheme. This would speed up each PWV measurement by a factor of 5 and delivering data with a temporal resolution of 5 ms. Preliminary experiments however showed, that these data still are exposed to significant fluctuations and inaccuracies with the need of applying further temporal low pass filtering. k-t BLAST acceleration in combination with the semi-automatic segmentation however prevents the need of additional temporal filtering and was therefore selected to be the preferred choice to accelerate the data acquisition.

In the present paper, a 2D-multi-slice approach was preferred instead of using a 3D approach. Thus, the measurement time for one PWV data point could be kept by about 1 min. Keeping in mind that a constant heart rate is crucial for the accurate measurement of the blood flow and the cross sectional area, the short measurement time is assumed to add to the robustness of the PWV calculation. Moreover, it is now feasible to estimate local elastic properties at many distinct locations at the aorta in between one measurement session. When manually segmenting cross secional areas of the aortic vessel for each dataset however, this would lead to a high work load for the operator meaning hours of processing data for just 10 different PWV datasets. To facilitate data processing and to enhance data reliability we introduced a semi-automatic approach for segmenting cross sectional areas of the vessel lumen. The results are similar to those observed with manual segmentation, but are corrected from any operator bias. When using the manual segmentation approach *κ*=0.38 was found for the intraobserver variability (*n*=10) and *κ*=0.28 for the interobserver variability (*n*=10). For the semi-automatic approach this operator bias can be avoided almost completely. Gotschy et al. could show in phantom experiments, that when using the manual segmentation approach local CMR-PWV-measures agree with PWV-values obtained by external non-CMR reference methods [26]. Thus, since the semi-automatic segmentation data matches with the manual segmentation, PWV-values obtained by the new proposed methods are assumed to provide accurate values.

The proposed measurement and postprocessing methods were applied in two small groups of 8-month old ApoE ^(−/−)^ and C57BL/6J-mice, respectively. As expected and also shown in previous studies, the total mean values for the PWV in each group are significantly different with elevated PWV values for the ApoE ^(−/−)^-group [20, 27]. However, the limited number of in vivo experiments, that should serve to prove the feasibility of the proposed method, did not allow to reveal any spatial patterns for the distribution of the local PWV. Especially in the upper part of the abdominal aorta, only a few data points could be obtained. The poor quality of the PC-CINE-data sets in this region can mainly be attributed to susceptibility artefacts in the proximity of the diaphragm. In the present study, a first order automatic shimming-routine was applied prior to each measurement. Thus, at lower field strengths and with an increased effort in shimming, it should be feasible to reduce susceptibility artefacts.

Yet the limited number of data-points showed a higher dispersion in the ApoE ^(−/−)^-group compared to the wild-type group. While these effects did not bear any deterministic spatial patterns, we assume that atherosclerotic effects on the vessel wall elasticity in the abdominal aorta are scattered more randomly along the vessel wall, in contrast to the existence of typical locations for lesion as for instance in the aortic arch [28].

PWV was examined over a range of 12 mm in the abdominal aorta. In humans, it could be shown that the PWV increases from 4–5 m/s in the ascending aorta to 5–6 m/s in the abdominal aorta [17, 29, 30]. This effect of increased PWV values from proximal to distal could not be confirmed with the current data. Since in humans the magnitude of this effect was about 20% from ascending to descending aorta, the smaller aortic section that was investigated in the present study is supposed to show less increase of the PWV. With the limited amount of data, this effect might be confounded by the individual error level for each PWV measurement.

## Conclusion

In summary, the in vivo experiments performed with the highly accelerated and automated new technique proposed in the present work could verify PWV values as presented in previous studies. Future work should focus on follow-up studies in the context of larger samples to potentially reveal patterns of spatial distribution of local elastic properties. In combination with the examination of local inflammatory processes and parameters describing the vessel wall mechanics, such as wall shear stress, the proposed technique would significantly add to the understanding of the pathogenesis of early atherosclerosis.

## Abbreviations

APOE: Apolipoprotein E
AS: Arterial stiffness
BLAST: Broad-use linear acquisition speed-up technique
CVD: Cardiovascular disease
ESRD: End-stage renal disease
FLASH: Fast low angle shot
CMR: Cardiovascular magnetic resonance imaging
PC: Phase contrast
PWV: Pulse wave velocity
QA: Flow/Area
TT: transit-time

## References

[1] Hansen TW, Staessen JA, Torp-Pedersen C, Rasmussen S, Thijs L, Ibsen H, Jeppesen J (2006). Prognostic value of aortic pulse wave velocity as index of arterial stiffness in the general population. Circulation.

[2] Mitchell GF, Hwang SJ, Vasan RS, Larson MG, Pencina MJ, Hamburg NM, Vita JA, Levy D, Benjamin EJ (2010). Arterial stiffness and cardiovascular events the framingham heart study. Circulation.

[3] Cavalcante JL, Lima JAC, Redheuil A, Al-Mallah MH (2011). Aortic stiffness: current understanding and future directions. J Am Coll Cardiol.

[4] Laurent S, Katsahian S, Fassot C, Tropeano AI, Gautier I, Laloux B, Boutouyrie P (2003). Aortic stiffness is an independent predictor of fatal stroke in essential hypertension. Stroke.

[5] Sutton-Tyrrell K, Najjar SS, Boudreau RM, Venkitachalam L, Kupelian V, Simonsick EM, Havlik R, Lakatta EG, Spurgeon H, Kritchevsky S (2005). Elevated aortic pulse wave velocity, a marker of arterial stiffness, predicts cardiovascular events in well-functioning older adults. Circulation.

[6] Pannier B, Guérin AP, Marchais SJ, Safar ME, London GM (2005). Stiffness of capacitive and conduit arteries prognostic significance for end-stage renal disease patients. Hypertension.

[7] Asmar R, Benetos A, Topouchian J, Laurent P, Pannier B, Brisac AM, Target R, Levy BI (1995). Assessment of arterial distensibility by automatic pulse wave velocity measurement validation and clinical application studies. Hypertension.

[8] Cruickshank K, Riste L, Anderson SG, Wright JS, Dunn G, Gosling RG (2002). Aortic pulse-wave velocity and its relationship to mortality in diabetes and glucose intolerance an integrated index of vascular function?. Circulation.

[9] Redheuil A, Yu WC, Wu CO, Mousseaux E, de Cesare A, Yan R, Kachenoura N, Bluemke D, Lima JA (2010). Reduced ascending aortic strain and distensibility earliest manifestations of vascular aging in humans. Hypertension.

[10] Vulliémoz S, Stergiopulos N, Meuli R (2002). Estimation of local aortic elastic properties with mri. Magn Reson Med.

[11] Meir KS, Leitersdorf E (2004). Atherosclerosis in the apolipoprotein e–deficient mouse a decade of progress. Arterioscler Throm Vas.

[12] Plump AS, Breslow JL (1995). Apolipoprotein e and the apolipoprotein e-deficient mouse. Annu Rev Nutr.

[13] Chatterjee S, Bedja D, Mishra S, Amuzie C, Avolio A, Kass DA, Berkowitz D, Renehan M (2014). Inhibition of glycosphingolipid synthesis ameliorates atherosclerosis and arterial stiffness in apolipoprotein e-/- mice and rabbits fed a high-fat and-cholesterol diet. Circulation.

[14] Hartley CJ, Reddy AK, Madala S, Martin-McNulty B, Vergona R, Sullivan ME, Halks-Miller M, Taffet GE, Michael LH, Entman ML, Wang YX (2000). Hemodynamic changes in apolipoprotein e-knockout mice. Am J Physiol Heart Circ Physiol.

[15] Parczyk M, Herold V, Klug G, Bauer WR, Rommel E, Jakob PM (2010). Regional in vivo transit time measurements of aortic pulse wave velocity in mice with high-field cmr at 17.6 tesla. J Cardiovasc Magn Reson.

[16] Zhao X, Pratt R, Wansapura J (2009). Quantification of aortic compliance in mice using radial phase contrast mri. J Magn Reson Imaging.

[17] Laurent S, Cockcroft J, Van Bortel L, Boutouyrie P, Giannattasio C, Hayoz D, Pannier B, Vlachopoulos C, Wilkinson I, Struijker-Boudier H (2006). European Network for Non-invasive Investigation of Large Arteries. Expert consensus document on arterial stiffness: methodological issues and clinical applications. Eur Heart J.

[18] Assemat P, Siu K, Armitage J, Hokke S, Dart A, Chin-Dusting J, Hourigan K (2014). Haemodynamical stress in mouse aortic arch with atherosclerotic plaques: Preliminary study of plaque progression. Comput Struct Biotechnol J.

[19] Ben-Dor I, Barbash IM, Aly O, Dvir D, Deksissa T, Okubagzi P, Torguson R, Lindsay J, Satler LF, Pichard AD (2013). Correlation of brain natriuretic peptide levels in patients with severe aortic stenosis undergoing operative valve replacement or percutaneous transcatheter intervention with clinical, echocardiographic, and hemodynamic factors and prognosis. Am J Cardiol.

[20] Gotschy A, Bauer E, Schrodt C, Lykowsky G, Ye YX, Rommel E, Jakob PM, Bauer WR, Herold V (2013). Local arterial stiffening assessed by mri precedes atherosclerotic plaque formation. Circ Cardiovasc Imaging.

[21] Herold V, Parczyk M, Mörchel P, Ziener CH, Klug G, Bauer WR, Rommel E, Jakob PM (2009). In vivo measurement of local aortic pulse-wave velocity in mice with mr microscopy at 17.6 tesla. Magn Reson Med.

[22] Winter P, Kampf T, Helluy X, Gutjahr FT, Meyer CB, Rommel E, Bauer WR, Jakob PM, Herold V (2013). Fast retrospectively triggered local pulse-wave velocity measurements in mice with cmr-microscopy using a radial trajectory. J Cardiovasc Magn Reson.

[23] Tsao J, Boesiger P, Pruessmann KP (2003). k-t blast and k-t sense: dynamic mri with high frame rate exploiting spatiotemporal correlations. Magn Reson Med.

[24] Kozerke S, Tsao J (2004). Reduced data acquisition methods in cardiac imaging. Top Magn Reson Imaging.

[25] Stadlbauer A, van der Riet W, Crelier G, Salomonowitz E (2010). Accelerated time-resolved three-dimensional MR velocity mapping of blood flow patterns in the aorta using SENSE and k-t BLAST. Eur J Radiol.

[26] Gotschy A, Bauer WR, Winter P, Nordbeck P, Rommel E, Jakob PM, Herold V (2017). Local versus global aortic pulse wave velocity in early atherosclerosis: An animal study in ApoE-/–mice using ultrahigh field MRI. PLoS ONE.

[27] Wang YX (2005). Cardiovascular functional phenotypes and pharmacological responses in apolipoprotein e deficient mice. Neurobiol Aging.

[28] Feintuch A, Ruengsakulrach P, Lin A, Zhang J, Zhou YQ, Bishop J, Davidson L, Courtman D, Foster FS, Steinman DA, Henkelman RM, Ethier CR (2007). Hemodynamics in the mouse aortic arch as assessed by mri, ultrasound, and numerical modeling. Am J Physiol Heart Circ Physiol.

[29] Nichols WW, ORourke MF (2005). McDonalds blood flow in arteries. Theoretical, Experimental and Clinical Principles. 5th ed.

[30] Latham RD, Westerhof N, Sipkema P, Rubal BJ, Reuderink P, Murgo JP (1985). Regional wave travel and reflections along the human aorta: a study with six simultaneous micromanometric pressures. Circulation.

